# Increasing the inspiratory time and I:E ratio during mechanical ventilation aggravates ventilator-induced lung injury in mice

**DOI:** 10.1186/s13054-015-0759-2

**Published:** 2015-01-28

**Authors:** Holger C Müller-Redetzky, Matthias Felten, Katharina Hellwig, Sandra-Maria Wienhold, Jan Naujoks, Bastian Opitz, Olivia Kershaw, Achim D Gruber, Norbert Suttorp, Martin Witzenrath

**Affiliations:** Department of Infectious Diseases and Pulmonary Medicine, Charité - Universitätsmedizin Berlin, Berlin, Germany; Department of Veterinary Pathology, Freie Universität Berlin, Berlin, Germany

## Abstract

**Introduction:**

Lung-protective ventilation reduced acute respiratory distress syndrome (ARDS) mortality. To minimize ventilator-induced lung injury (VILI), tidal volume is limited, high plateau pressures are avoided, and positive end-expiratory pressure (PEEP) is applied. However, the impact of specific ventilatory patterns on VILI is not well defined. Increasing inspiratory time and thereby the inspiratory/expiratory ratio (I:E ratio) may improve oxygenation, but may also be harmful as the absolute stress and strain over time increase. We thus hypothesized that increasing inspiratory time and I:E ratio aggravates VILI.

**Methods:**

VILI was induced in mice by high tidal-volume ventilation (HV_T_ 34 ml/kg). Low tidal-volume ventilation (LV_T_ 9 ml/kg) was used in control groups. PEEP was set to 2 cm H_2_O, FiO_2_ was 0.5 in all groups. HV_T_ and LV_T_ mice were ventilated with either I:E of 1:2 (LV_T_ 1:2, HV_T_ 1:2) or 1:1 (LV_T_ 1:1, HV_T_ 1:1) for 4 hours or until an alternative end point, defined as mean arterial blood pressure below 40 mm Hg. Dynamic hyperinflation due to the increased I:E ratio was excluded in a separate group of animals. Survival, lung compliance, oxygenation, pulmonary permeability, markers of pulmonary and systemic inflammation (leukocyte differentiation in lung and blood, analyses of pulmonary interleukin-6, interleukin-1β, keratinocyte-derived chemokine, monocyte chemoattractant protein-1), and histopathologic pulmonary changes were analyzed.

**Results:**

LV_T_ 1:2 or LV_T_ 1:1 did not result in VILI, and all individuals survived the ventilation period. HV_T_ 1:2 decreased lung compliance, increased pulmonary neutrophils and cytokine expression, and evoked marked histologic signs of lung injury. All animals survived. HV_T_ 1:1 caused further significant worsening of oxygenation, compliance and increased pulmonary proinflammatory cytokine expression, and pulmonary and blood neutrophils. In the HV_T_ 1:1 group, significant mortality during mechanical ventilation was observed.

**Conclusion:**

According to the “baby lung” concept, mechanical ventilation-associated stress and strain in overinflated regions of ARDS lungs was simulated by using high tidal-volume ventilation. Increase of inspiratory time and I:E ratio significantly aggravated VILI in mice, suggesting an impact of a “stress/strain × time product” for the pathogenesis of VILI. Thus increasing the inspiratory time and I:E ratio should be critically considered.

**Electronic supplementary material:**

The online version of this article (doi:10.1186/s13054-015-0759-2) contains supplementary material, which is available to authorized users.

## Introduction

Mechanical ventilation is a life-saving intervention for patients with acute respiratory failure without alternative. However, mechanical ventilation itself can aggravate or even initiate lung injury, termed ventilator-induced lung injury (VILI) [1]. The impact of VILI on mortality and morbidity in acute respiratory distress syndrome (ARDS) patients is evident. Lung-protective ventilation strategies have been implemented to minimize VILI, consisting of limitation of tidal volumes (V_T_) to 6 ml/kg body weight, use of positive end-expiratory pressure (PEEP), and avoidance of plateau pressures above 30 cmH_2_O [2,3]. Further, even previously healthy lungs seem to benefit from lung-protective mechanical ventilation, and low tidal-volume ventilation causes an inflammatory response in healthy lungs [4]. Moreover, functional residual capacity is considerably reduced in ARDS (“baby lung concept”), and therefore, ventilated areas of the ARDS lung encounter dramatically increased transparenchymal forces, even under low tidal-volume ventilation. Thus, a certain safety threshold for VILI does not seem to exist, and any effort to minimize VILI further might be of relevance, particularly for the most severely ill ARDS patients [5].

Of note, little is known regarding the impact of ventilator adjustments on VILI. The absolute inspiratory lung strain, which is defined as the end-inspiratory transpulmonary pressure, and the absolute lung strain, defined as V_T_/FRC, are central drivers of VILI [5]. We hypothesized that, in addition, the duration of lung stress and strain is relevant, proposing a Time × Stress/strain product that affects VILI.

Increasing the inspiration-to- expiration ratio (I:E) and thereby the inspiratory time (t_i_) of the respiratory cycle can improve oxygenation. The two main underlying mechanisms are probably prolonged gas exchange during inspiration in lung areas that do not take part in gas exchange during expiration, and recruitment of lung tissue due to increased intrinsic PEEP generated by dynamic hyperinflation [6,7]. It is tempting to use this intervention to improve oxygenation at bedside as, despite all efforts made to stabilize an appropriate residual volume by titrating PEEP, almost always, recruitable lung regions remain. Conversely, increasing I:E will result in an increased Time × Stress/strain product that might aggravate VILI. A previous experimental study [8] and a recently published review of numerous animal models of VILI underscore this hypothesis [9].

In this study, we therefore investigated the impact of I:E on VILI in an experimental VILI mouse model and found that an increased I:E ratio significantly aggravates VILI in mice, suggesting the relevance of a role of a Stress/strain × Time in the pathogenesis of VILI.

## Material and methods

### Ethics statement

All animal experiments were approved by institutional (Charité-Universitätsmedizin Berlin) and governmental (Landesamt für Gesundheit und Soziales Berlin; G 0130/12) authorities.

### Mice

Female C57BL/6 mice (8 to 10 weeks; 18 to 20 g; Charles River, Sulzfeld, Germany) were used.

### Mechanical ventilation

Mice were anesthetized with intraperitoneal injections of fentanyl (75 μg/kg), midazolam (1.5 mg/kg), and medetomedin (0.75 mg/kg). Repetitively, fentanyl (16 μg/kg), midazolam (0.33 mg/kg), and medetomedin (0.16 mg/kg) were supplied via an intraperitoneal catheter, when required, to guarantee adequate anesthesia during the experiment. Body temperature was maintained at 37°C by a body temperature-controlled heating pad. Mice were tracheotomized, intubated, and ventilated with low tidal-volume (LV_T_) respirator settings (tidal volume of 9 ml/kg, respiratory rate of 160 per min^ute^, I:E ratio of 1:2, FiO_2_ of 0.5). A carotid artery catheter was placed for blood pressure monitoring and infusion of a balanced electrolyte solution (Jonosteril; Fresenius Kabi, Bad Homburg Germany) containing 150 *M* trometamolhydrochloride (350 μl/h).

A urinary catheter was inserted. Mean arterial blood pressure, heart rate, peripheral oxygen saturation (MouseOx; Starr Life Science Corp., Pittsburgh, PA, USA) and urine output were measured. Mice were ventilated by using a special rodent ventilation system, which continuously recorded airway pressure, respiratory rate, and tidal volume (flexiVent; Scireq, Montreal, QC, Canada). After preparation, a recruitment maneuver was performed (increasing of the airway pressure to 30 cmH_2_O), and mice were ventilated for 4 hours with the following ventilator settings:

#### Low tidal-volume (LV_T_) groups

Mice were ventilated with a tidal volume of 9 ml/kg, respiratory rate of 160 per minute, and I:E ratio of either 1:2 or 1:1 (LV_T_ 1:2; LV_T_ 1:1). A deep inspiration (30 cmH_2_O for 1 second), was performed every 10 minutes, in addition to the applied positive end-expiratory pressure (PEEP) to avoid atelectasis. Notably, this protocol does not cause measurable lung injury in mice [10].

#### High tidal-volume (HV_T_) groups

Mice were ventilated with a tidal volume of 34 ml/kg, respiratory rate of 70 per minute, and I:E ratio of 1:2 or 1:1, respectively (HV_T_ 1:2; HV_T_ 1:1).

**Figure 1:**
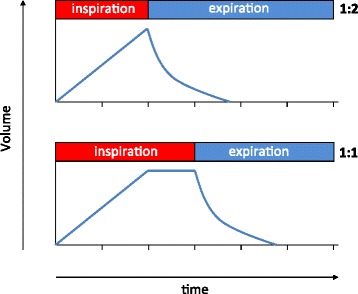
**Schematic graphic of the respiratory cycle during I:E ratio of 1:2 and 1:1.** In all I:E 1:1 groups, the inspiratory time was prolonged by adding an inspiratory hold after completion of lung inflation, thereby leaving pressure and flow acceleration during inspiration identical between the corresponding LV_T_ and HV_T_ 1:2 groups.

In all I:E 1:1 groups, the inspiratory time was prolonged by adding an inspiratory hold after completion of lung inflation, thereby leaving pressure and flow acceleration during inspiration identical between the corresponding LV_T_ and HV_T_ 1:2 groups, as schematically illustrated in Figure 1.

To generate baseline values at the beginning of mechanical ventilation, a group of mice referred to as nonventilated control (ctr) was anesthetized and operated as outlined earlier. After an identical recruitment maneuver, ctr mice were ventilated for 5 minutes with adjustments identical to those of the LV_T_ 1:2 mice. After measurement of baseline lung functions and hemodynamics, the experiment was terminated.

PEEP of 2 cmH_2_O and an FiO_2_ of 0.5 were applied throughout the experiments in all LV_T_ and HV_T_ groups. After 235 minutes of mechanical ventilation (MV), the I:E ratio was switched to 1:2 in all ventilated groups, and the inspired oxygen fraction was increased to an FiO_2_ of 1.0. After 240 minutes of MV, mice were killed by rapid exsanguination via the carotid artery catheter.

An alternative end point was defined as decrease of mean arterial blood pressure below 40 mm Hg, as this safely predicts death in this model. The I:E ratio was then switched to 1:2, and the inspired oxygen fraction was increased to an FiO_2_ of 1.0. After a further 5 minutes of MV, mice were killed by exsanguination via the carotid artery catheter.

To exclude dynamic hyperinflation in the HV_T_ 1:1 group, an additional set of animals was ventilated according to the HV_T_ 1:2 ventilation pattern for 30 minutes, and then the I:E ratio was increased to 1:1 for 30 minutes. This procedure was repeated. Mean airway pressure and dynamic compliance were recorded.

### Lung function

After the initial recruitment maneuver, dynamic elastance, resistance, and compliance were measured by using a forced oscillation technique. Measurements were repeated every 10 minutes throughout the experiment. In addition, static-compliance values were determined after exsanguination.

### Blood gas analyses

Blood samples were analyzed for p_a_O_2_ with a blood-gas analyzer (ABL-800; Radiometer, Copenhagen, Denmark). The P/F ratio was calculated as P/F = p_a_O_2_/FiO_2_. Oxygenation Index was calculated as OI = mean airway pressure × FiO_2_/p_a_O_2_.

### Lung permeability

After exsanguination, the left-stem bronchus was ligated, and bronchoalveolar lavage (BAL) of the right lung was performed twice with 400 μl PBS each. From each BAL fluid (BALF) portion, 250 μl was pooled, and BALF albumin concentration, as well as plasma albumin concentration, were determined with ELISA (Bethyl Laboratories Inc., Montgomery, AL, USA). Permeability was assessed by calculating the albumin BALF/plasma ratio.

### qRT-PCR

Lungs were flushed. RNA was extracted with TRIzol (Ambion; Life Technologies, Darmstadt, Germany) treatment of lung homogenates and reverse-transcribed by using a high-capacity reverse transcription kit (Applied Biosystems, Life Technologies, Darmstadt, Germany). Quantitative PCR (qRT-PCR) was performed on ABI 7300 by using TaqMan gene-expression assays (Applied Biosystems). The PCR conditions included initial denaturation in one cycle of 2 minutes at 50°C and 10 minutes at 95°C, followed by 40 cycles of 15 seconds at 95°C, 20 seconds at 60°C, and 1 minute at 72°C. The input was normalized to the average expression of GAPDH. Primer and probe sequences are provided in Table S1 in Additional file 1.

### Leukocytes in BAL fluid and blood

Leukocytes in BALF were differentiated with flow cytometry, according to their side-scatter/forward-scatter characteristics, and CD45, Gr-1, and F4-80 expression (FACSCalibur; BD, Heidelberg, Germany). Blood leukocytes were quantified and differentiated with flow cytometry by using TruCount-Tubes according to cellular side-scatter/forward-scatter characteristics and CD45, Gr-1, and CD3 expression.

### Quantification of cytokines

Cytokines were quantified from total protein of flushed and homogenized left lungs and from plasma samples by using the multiplex cytokine assay technique (BioRad, Hercules, CA, USA).

### Histopathology

Lung samples were fixed in 4% formaldehyde solution and routinely embedded in paraffin. The 5-μm-thick sections were cut, dewaxed, and stained with hematoxylin and eosin (H&E) or periodic acid-Schiff (PAS). Histopathology was performed by two European College of Veterinary Pathologists (ECVP) board-certified pathologists, who were blinded to the study groups.

### Data analyses

Data are expressed as box-and-whisker plots, or columns (mean ± SEM). For comparison between groups, a Mann–Whitney *U* test was performed. *P* values <0.05 were considered statistically significant. For survival analyses, a log rank test was applied.

## Results

### Increasing inspiratory time and I:E ratio did not result in dynamic hyperinflation

To rule out relevant dynamic hyperinflation, HV_T_ animals were ventilated with an alternating I:E ratio (1:2 versus 1:1) in 30-minute intervals for 120 minutes, and mean airway pressure and dynamic compliance (C_dyn_) were measured. Mean airway pressure remained stable during the specific interval of MV (see Figure S1A in Additional file 2). The higher but stable mean airway pressure during HV_T_ 1:1 is explained by the increased inspiratory time during HV_T_ 1:1 (see Figure S1A in Additional file 2). C_dyn_ remained stable during MV, irrespective of the adjusted I:E ratio (see Figure S1B in Additional file 2). Thus, HV_T_ 1:1 ventilation did not lead to relevant dynamic hyperinflation.

### Increasing the inspiratory time and I:E ratio during MV increased mortality in VILI

**Figure 2:**
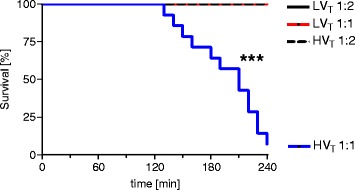
**Increasing the inspiratory time and I:E ratio during MV increased mortality in VILI.** Mice were mechanically ventilated for 4 hours with either low tidal volume (LV_T_ 9 ml/kg) or high tidal volume (HV_T_ 34 ml/kg) and an inspiratory/expiratory ratio of 1:2 or 1:1, respectively. If the mean arterial pressure decreased below 40 mm Hg, the experiment was prematurely terminated, as this predicts death with certainty in this model. *n* = 13-14 each group; ***P < 0.001.

All mice of the low tidal-volume groups ventilated with an I:E ratio of either 1:2 or 1:1 (LV_T_ 1:2; LV_T_ 1:1) and of the high tidal-volume group ventilated with an I:E ratio of 1:2 survived the procedures. Increasing the I:E ratio in the HV_T_ group to 1:1 resulted in premature termination of the experiment in 13 of 14 mice because of dropping of mean arterial blood pressure below 40 mm Hg (alternative end point), corresponding to a 92.1% mortality in the HV_T_ 1:1 group (Figure 2).

### Increasing the inspiratory time und I:E ratio increased lung injury

**Figure 3:**
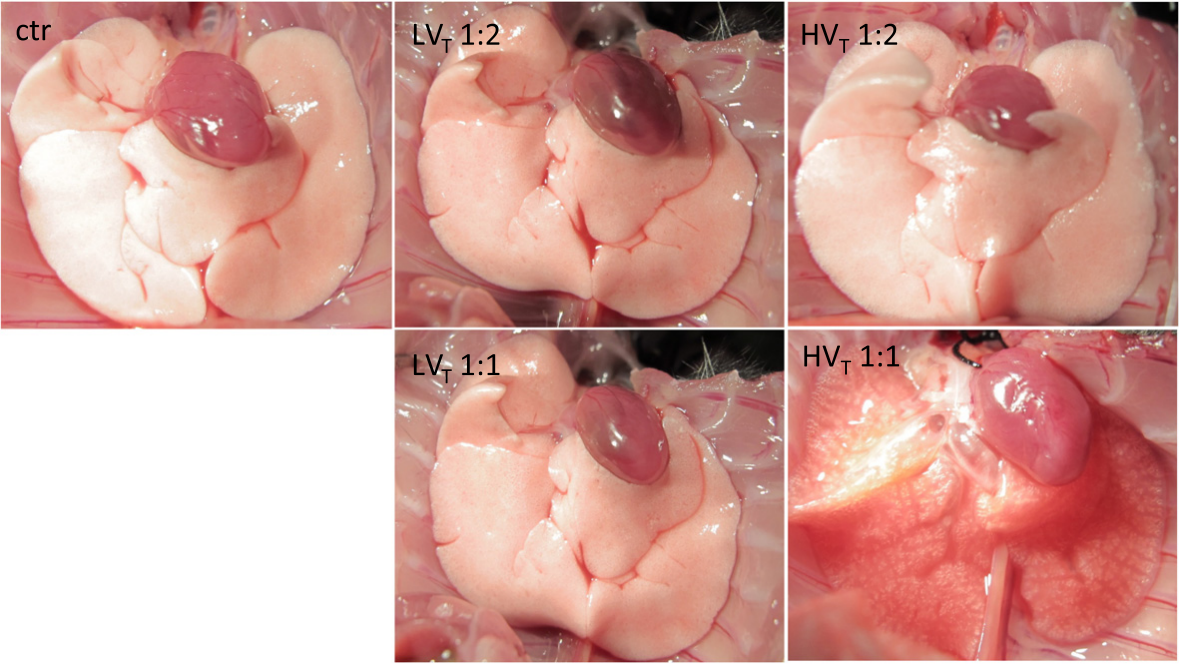
**Increasing the inspiratory time and I:E ratio during MV increased VILI.** Mice were mechanically ventilated for 4 hours with either low tidal volume (LV_T_ 9 ml/kg) or high tidal volume (HV_T_ 34 ml/kg) and an inspiratory/expiratory ratio of 1:2 or 1:1, respectively. An alternative end point was defined as decreasing of mean arterial blood pressure below 40 mm Hg, as this predicts death with certainty in this model. Controls (ctr) were subjected to LV_T_ 1:2 ventilation only during operation and were killed before the 4-hour ventilation protocol started. Ctr and LV_T_ groups showed no signs of lung injury macroscopically. HV_T_ 1:2 revealed only subtle macroscopic signs of injury, whereas the HV_T_ 1:1 group showed massive edema formation. Representative images from 13 to 14 animals per group are shown.

Lungs from ctr, LV_T_ 1:2, and LV_T_ 1:1 showed no macroscopic or histologic signs of lung injury. HV_T_ 1:2 ventilated mice exhibited significant histopathologic signs of lung injury, whereas only distinct signs of injury were seen macroscopically. HV_T_ 1:1 led to dramatic macroscopic and histopathologic lesions. In both HV_T_ groups but not in the controls, the lung architecture was compromised by severe alveolar collapse and emphysema. Histopathology revealed severe perivascular edema, damage of the alveolar walls with desquamation of alveolar epithelial cells type I and formation of hyaline membranes, increasing numbers of intraalveolar cells (neutrophils and macrophages) and occasional necrosis of bronchiolar epithelium. Severe lung lesions were observed histologically on HE-stained tissues, with no differences seen between both groups (Figure 3 and Figure S2 in Additional file 3).

**Figure 4:**
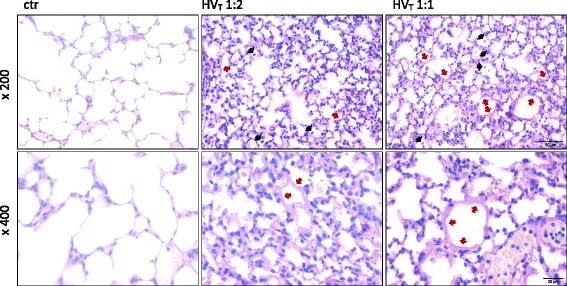
**Increasing the inspiratory time and I:E ratio during MV increased histopathologic signs of lung injury.** Mice were mechanically ventilated for 4 hours with either low tidal volume (LV_T_ 9 ml/kg) or high tidal volume (HV_T_ 34 ml/kg) and an inspiratory/expiratory ratio of 1:2 or 1:1, respectively. An alternative end point was defined as decreasing of mean arterial blood pressure below 40 mm Hg, which predicts death with certainty in this model. Controls (ctr) were subjected to LV_T_ 1:2 ventilation only during operation and were killed before the 4-hour ventilation protocol started. Histopathology of lungs from ctr, HV_T_ 1:2, and HV_T_ 1:1 groups, stained with the periodic acid-Schiff (PAS) reaction, are shown: In contrast to the ctr group, both ventilated groups had damage of the alveolar walls with septal thickening, necrosis, and desquamation of alveolar epithelial cells type I, formation of hyaline membranes (red arrows), and increased numbers of intraalveolar cells (predominantly neutrophils and macrophages, black arrows). PAS reaction highlighted the more severe and more continuous as well as thicker hyaline membranes along the alveolar surfaces of lungs from the HV_T_ 1:1 group. Top panel: magnification × 200; Bottom panel: magnification × 400. Representative images from each group (*n* = 4 each) are shown.

**Figure 5:**
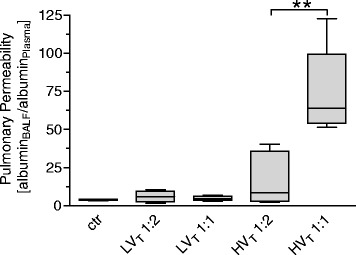
**Increasing the inspiratory time and I:E ratio during MV increased pulmonary permeability in VILI.** Mice were mechanically ventilated for 4 hours with either low tidal volume (LV_T_ 9 ml/kg) or high tidal volume (HV_T_ 34 ml/kg) and an inspiratory/expiratory ratio of 1:2 or 1:1, respectively. An alternative end point was defined as decreasing of mean arterial blood pressure below 40 mm Hg, which predicts death with certainty in this model. Controls (ctr) were subjected to LV_T_ 1:2 ventilation only during operation and were killed before the 4-hour ventilation protocol started. Albumin concentrations in bronchoalveolar lavage fluid (BALF) and plasma were determined. An increased albumin BALF/plasma ratio indicated enhanced lung permeability. *n* = 5 to 6 each group; ** < 0.01.

Periodic acid-Schiff (PAS) reaction clearly visualized hyaline membranes diffusely distributed throughout the lung parenchyma, indicative of marked damage of the alveolar membrane. However, in HV_T_ 1:2 lungs, hyaline membranes appeared only occasionally as continuous thin layers on the alveolar surface, while lungs of the HV_T_ 1:1 group had thicker hyaline membranes, which commonly completely covered the surface of dilated alveoli (Figure 4). Because pulmonary vascular leakage is a hallmark of ARDS and VILI, we quantified lung permeability by measuring the albumin concentration in bronchoalveolar lavage fluid (BALF) and plasma and by calculating the BALF/plasma albumin ratio. Compared with ctr mice, LV_T_ 1:2 and LV_T_ 1:1 did not result in increased permeability. In contrast, HV_T_ 1:2 mice showed a trend toward increased permeability compared with ctr and LV_T_ groups, whereas HV_T_ 1:1 evoked a dramatic increase in pulmonary vascular permeability (Figure 5).

### Increasing the inspiratory time and I:E ratio reduced oxygenation capacity in VILI

**Figure 6:**
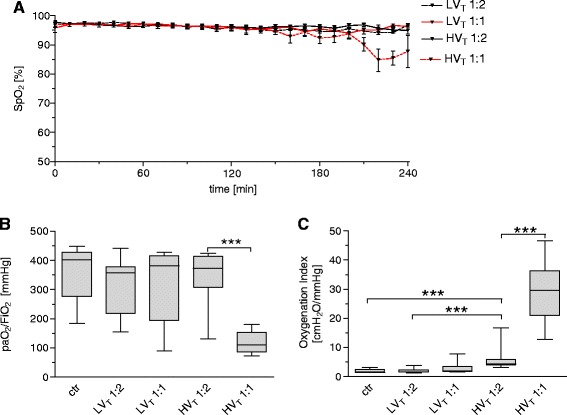
**Increasing the inspiratory time and I:E ratio reduced oxygenation capacity in VILI.** Mice were mechanically ventilated for 4 hours with either low tidal volume (LV_T_ 9 ml/kg) or high tidal volume (HV_T_ 34 ml/kg) and an inspiratory/expiratory ratio of 1:2 or 1:1, respectively. An alternative end point was defined as decreasing of mean arterial blood pressure below 40 mm Hg, which predicts death with certainty in this model. Controls (ctr) were subjected to LV_T_ 1:2 ventilation only during operation and were killed before the 4-hour ventilation protocol started. **(A)** Pulse oximetry revealed stable oxygen saturation in LV_T_ 1:2, LV_T_ 1:1, and HV_T_ 1:2 groups, whereas the HV_T_ 1:1 ventilated mice developed a decrease of oxygen saturation during the 4-hour ventilation period. End-point measurements of arterial partial pressure of oxygen were performed, and the P/F ratio **(B)**, and the oxygenation index were calculated **(C)**. *n* = 13 to 14 in each group; ***P < 0.001.

Peripheral oxygen saturation was measured continuously throughout the experiment. Partial pressure of oxygen in arterial blood and mean airway pressure were measured at the end of the experiment, and P/F ratio as well as oxygenation index (OI) were calculated. Whereas LV_T_ 1:2, LV_T_ 1:1, and HV_T_ 1:2 groups showed stable oxygenation regarding SpO_2_ and P/F throughout the experiment (Figure 6A,B), the oxygenation index implied a reduced oxygenation capacity in HV_T_ 1:2 mice compared to ctr and LV_T_ groups (Figure 6C). HV_T_ 1:1 resulted in severe impairment of oxygenation compared with ctr, LV_T_, and HV_T_ 1:2 groups (Figure 6A to C).

### Increasing the inspiratory time and I:E ratio deteriorated lung function in VILI

**Figure 7:**
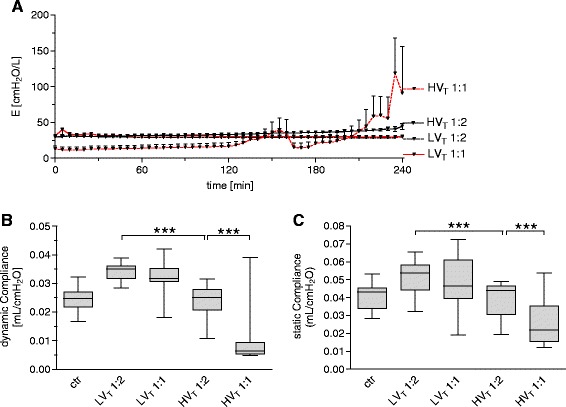
**Increasing the inspiratory time and I:E ratio deteriorated lung function in VILI.** Mice were mechanically ventilated for 4 hours with either low tidal volume (LV_T_ 9 ml/kg) or high tidal volume (HV_T_ 34 ml/kg) and an inspiratory/expiratory ratio of 1:2 or 1:1, respectively. An alternative end point was defined as decreasing of mean arterial blood pressure below 40 mm Hg, which predicts death with certainty in this model. Controls (ctr) were subjected to LV_T_ 1:2 ventilation only during operation and were killed before the 4-hour ventilation protocol started. **(A)** Dynamic elastance (E) was measured every 10 minutes during the experiment. Results of end-point measurements of dynamic compliance **(B)** and static compliance **(C)** are shown. *n* = 13 to 14 in each group; ***P < 0.001.

Dynamic elastance was quantified every 10 minutes. While both LV_T_ groups showed stable elastance during the experiment, a slight increase over time in the HV_T_ 1:2 group, and a strong increase in the HV_T_ 1:1 group were observed (Figure 7A). Dynamic and static compliance at the respective end points of the experiment showed impaired compliance in the HV_T_ 1:2 compared with LV_T_ 1:2 mice. HV_T_ 1:1 led to a dramatic decrease in lung compliance (Figure 7B,C).

### Increasing the inspiratory time and I:E ratio: impact on hemodynamics and markers of tissue perfusion

HV_T_ animals were ventilated with an alternating I:E ratio (1:2 versus 1:1) in 30-minute intervals for 120 minutes, and mean arterial blood pressure was measured. Changing of the I:E ratio had no impact on mean arterial blood pressure (see Figure S3A in Additional file 4). In animals ventilated for 4 hours (LV_T_ 1:2, LV_T_ 1:1, HV_T_ 1:2, and HV_T_ 1:1), cumulative urine output and blood lactate levels at the respective experimental end points were quantified. No difference in urine output between the groups was evident, whereas HV_T_ 1:1 revealed slightly higher lactate levels than the HV_T_ 1:2 group (see Figure S3 B, C in Additional file 4).

### Increasing the inspiratory time and I:E ratio exacerbated the inflammatory response in VILI

**Figure 8:**
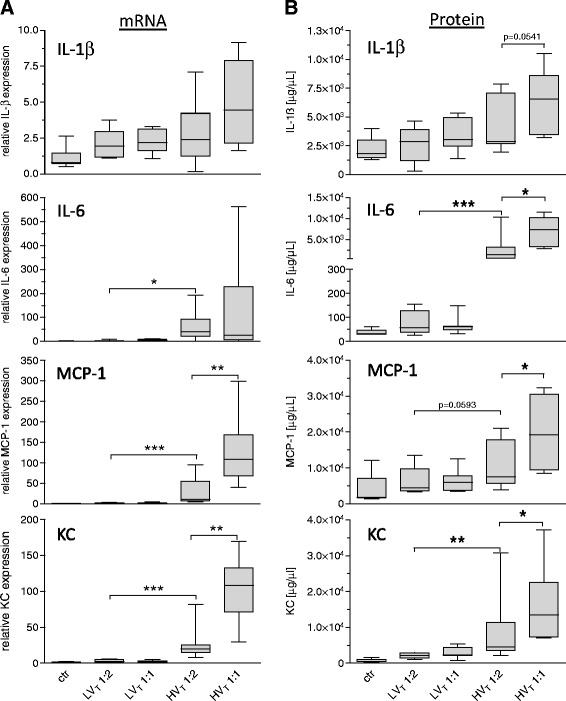
**Increasing the inspiratory time and I:E ratio increased the production of proinflammatory cytokines in VILI.** Mice were mechanically ventilated for 4 hours with either low tidal volume (LV_T_ 9 ml/kg) or high tidal volume (HV_T_ 34 ml/kg) and an inspiratory/expiratory ratio of 1:2 or 1:1, respectively. An alternative end point was defined as decreasing of mean arterial blood pressure below 40 mm Hg, which predicts death with certainty in this model. Controls (ctr) were subjected to LV_T_ 1:2 ventilation only during operation and were killed before the 4-hour ventilation protocol started. **(A)** mRNA levels of interleukin (IL)-1β, IL-6, macrophage chemotactic protein (MCP)-1, and keratinocyte-derived cytokine (KC) were measured with quantitative reverse transcription polymerase chain reaction in lung homogenates and normalized to GAPDH levels. **(B)** Protein levels of IL-1 β , IL-6, MCP-1, and KC were determined in lung homogenates by multiplex ELISA technique. *n* = 6 to 8 each group; *P < 0.05, **P < 0.01, ***P < 0.001.

We measured transcription of the proinflammatory cytokines IL-1β, IL-6, KC, and MCP-1 (Figure 8A) and protein concentrations of IL-1β, IL-6, KC, and MCP-1 in lung homogenates (Figure 8B). HV_T_ 1:2 increased IL-1β, IL-6, KC, and MCP-1 mRNA expression compared with ctr and LV_T_ mice. Expression of most of these proinflammatory mediators was further increased in the HV_T_ 1:1.

**Figure 9:**
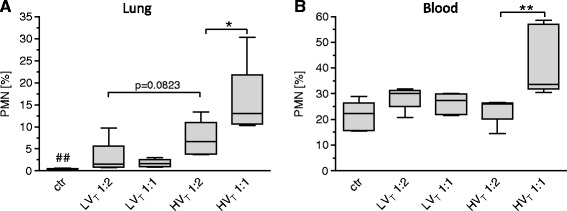
**Increasing the inspiratory time and I:E ratio increased number of pulmonary and blood neutrophils in VILI.** Mice were mechanically ventilated for 4 hours with either low tidal volume (LV_T_ 9 ml/kg) or high tidal volume (HV_T_ 34 ml/kg) and an inspiratory : expiratory ratio of 1:2 or 1:1, respectively. An alternative end point was defined as decreasing of mean arterial blood pressure below 40 mm Hg, which predicts death with certainty in this model. Controls (ctr) were subjected to LV_T_ 1:2 ventilation only during operation and were illed before the 4-hour ventilation protocol started. The fractions of neutrophils among leukocytes in the BALF **(A)** and in the blood **(B)** at the end point of the experiment are shown. *n* = 5 to 6 each group; *P < 0.05, **P < 0.01, ##*P* < 0.01 versus all.

LV_T_ 1:2 and LV_T_ 1:1 resulted in a certain increment of BALF neutrophils compared with ctr mice. In line with the elevation of proinflammatory cytokines in the lung, HV_T_ 1:2 led to a further increase of neutrophils, whereas HV_T_ 1:1 was associated with a significant neutrophil infiltration of the alveolar space (Figure 9A).

Furthermore, HV_T_ 1:1 exclusively resulted in an increased number of blood neutrophils, indicating a systemic inflammatory response (Figure 9B).

## Discussion

We provide strong evidence that increasing the I:E ratio during mechanical ventilation can aggravate VILI, indicating that not only the absolute lung stress and strain but also the time in which the lung is exposed to stress and strain (the Time × Stress and strain product) may affect the harm of mechanical ventilation.

VILI impairs survival of ARDS patients [2,3]. Besides cyclic opening and closing of lung during tidal ventilation, high airway pressures and high tidal volumes have been identified as main drivers of VILI. More precisely, not absolute airway pressure but the transpulmonary pressure termed lung stress, and not the absolute tidal volume, but its relation to the FRC, termed lung strain, are mechanical determinants of VILI [5]. Besides the amount of lung opening and closing that is correlated with ARDS mortality [11], the concept of intraparenchymal stress raisers during mechanical ventilation may have significant impact on the development of lung injury due to mechanical ventilation in the ARDS patient [5,12].

Recent clinical trials revealed that VILI is particularly relevant in patients with severe ARDS, and therefore, optimizing our ventilation strategies especially for those patients is desirable [13,14].

Lung stress and strain are not equally distributed throughout the respiratory cycle under MV, obviously being higher during inspiration than during expiration. The current study now provides evidence that not only absolute lung stress and strain but also increasing lung stress and strain in relation to the cycle time by prolonging the inspiratory time (t_i_) and increasing the I:E ratio aggravate VILI. This is in line with the theory of weighted lung strain during MV by Carioni and colleagues [9].

Physical forces during mechanical ventilation are sensed by the lung and induce a biochemical response characterized by inflammation and endoepithelial permeability, referred to as biotrauma [1,15,16]. Therefore we assessed lung permeability, detailed lung histology and markers of pulmonary inflammation. Even after the short observational time of 4 hours, we detected a significant impact of the increased t_i_ and I:E ratio on pulmonary cytokine levels, pulmonary neutrophil influx, systemic neutrophil counts, lung permeability, and histological signs of lung injury. KC and MCP-1 are chemotactic signals for neutrophils, which contribute to the development of VILI [17,18]. IL-1β was shown to induce endothelial permeability and aggravate VILI [19]. IL-6 is upregulated under mechanical ventilation and, although its exact role in VILI remains controversial, IL-6 levels are correlated with organ failure and outcome in ARDS [20-23]. Considering our findings, it is tempting to speculate that not only the absolute physical force or stretch but also its duration is sensed and responded to by the ventilated lung. This implies that mechanotransduction is increased, although the absolute amount of energy added to the system is kept constant, as pressure and volume remain unchanged throughout the inspiratory hold in the HV_T_ 1:1 group.

To test the hypothesis of this study, we implemented severely injurious ventilation in mice. The tidal volume of 34 ml/kg was extraordinarily high compared with the standard of lung-protective ventilation with 6 ml/kg in humans with ARDS. At first view, this might outrange the stress and strain applied during MV in ARDS patients. However, residual capacity in ARDS lungs is severely reduced, which is referred to as the “baby lung” of ARDS patients [24]. Notably, the sicker the patient and the lower the oxygenation capacity becomes, the greater is the reduction of the residual capacity and the intention to increase the relative portion of inspiratory time to improve oxygenation. As intensivists do not routinely quantify residual capacity at the bedside, we do not know how much lung strain is generated during MV, despite limiting the tidal volume to 6 ml/kg, especially in very severe ARDS. Further, ARDS is characterized by a high grade of tissue inhomogeneity, in which open, atelectatic, and collapsed but recruitable lung areas coexist, which locally results in lung stress exceeding the measured airway pressure by far [5].

Loss and inactivation of surfactant, a hallmark in ARDS, further aggravate local trauma [1]. Thus, applying high tidal volumes in healthy mouse lungs constitutes a reasonable experimental approach. Further, the currently used model of VILI meets the ATS criteria on lung injury in animals, including the evidence of inflammation, microscopic tissue injury, alteration of alveolar barrier function, and impaired oxygenation [25]. Vice versa, the observation that prolonging t_i_ in the LV_T_ groups did not increase detectable lung injury in healthy mice does not argue for the safety of an I:E ratio increase in lung-protective ventilation of ARDS patients.

In this study, it was highly important to control properly factors that might significantly bias the results. (a) Dynamic hyperinflation would increase intrinsic PEEP, which consecutively enhances residual volume and shifts tidal volume upward on the pressure/volume curve, eventually above the upper inflection point, resulting in augmented absolute lung stress and strain. Thus, we adjusted respiratory rates in both HV_T_ groups to 70 per minute to exclude dynamic hyperinflation in the HV_T_ I:E 1:1 group. (b) To keep dynamics of lung inflation identical between 1:1 and 1:2 groups, we prolonged t_i_ by adding an inspiratory hold. This excluded that a difference in pressure acceleration during inspiration or a difference of the total inflation time biased the results of the study. (c) Expiration was most probably similar in the HV_T_ 1:1 and HV_T_ 1:2, and in the LV_T_ 1:1 and LV_T_ 1:2 groups, respectively. Expiration is a passive process starting after the opening of the expiratory valve with end-inspiratory pressure being the driving force, which was similar in the respective groups. As dynamic hyperinflation could be excluded, exhalation was complete. (d) Respiratory rate, PEEP and FiO_2_ were identical in the HV_T_ and the LV_T_ groups, respectively and anesthesia and operation procedures were identical in all groups.

Intrathoracic pressure directly affects cardiac function (for an excellent review, see [26,27]), and increased intrathoracic pressure due to increased I:E ratio may decrease cardiac output [28,29]. Reduction of venous return seems to be the central mechanism reducing cardiac output by high intrathoracic pressure, which can be ameliorated by sufficient fluid support. In our study, mice received liberal fluid support to minimize reduction of cardiac output. Blood pressure and urine output were not affected by increased I:E ratio. Nevertheless, lactate levels were slightly elevated in HV_T_ 1:1 compared with HV_T_ 1:2 mice. Thus a certain reduction of cardiac output cannot be excluded. However, circulatory failure and resultant shock as cause of the premature death of the HV_T_ 1:1 animals would have resulted in significantly higher lactate levels, lower blood pressure, and particularly in reduction of urine output.

Thus the data provided here exclude profound hemodynamic deterioration as the underlying mechanism for the devastating outcome of the HV_T_ 1:1 group.

Although the data are conclusive and the results are clear, one can only speculate whether the findings can be translated to patients with ARDS. However, the study clearly emphasizes that for further improvement of lung-protective ventilation strategies, a deeper understanding of central factors for VILI is mandatory. In this regard, experimental studies like the present one are essential.

## Conclusion

The study design applied aimed to provide a proof of concept. The data show that beyond stress and strain, the time in which the lung is exposed to stress and strain (the Time × Stress and strain product) has dramatic impact on VILI. Therefore, it seems reasonable to minimize the Time ×  Strain product during MV. Particularly, increasing the I:E ratio should be critically revised in patients with ARDS.

## Key messages

- Increasing inspiratory time und thereby the I:E ratio aggravates VILI.
- Beyond stress and strain, the time during which the lung is exposed to stress and strain (the Time × Stress and strain product) has dramatic impact on VILI.

## Additional files

Additional file 1: Table S1. Providing primer and probe sequences used for qRT-PCR. Additional file 2: Figure S1. Giving mean airway pressure and dynamic compliance measurements under alternating I:E ratios (1:2 and 1:1) during HV_T_ ventilation. Additional file 3: Figure S2. Providing HE images of all experimental groups. Additional file 4: Figure S3. Providing hemodynamic data.

## Abbreviations

ARDS: Acute respiratory distress syndrome
BAL: bronchoalveolar lavage
BALF: bronchoalveolar lavage fluid
C_dyn_: dynamic compliance
Ctr: control
ELISA: enzyme-linked immunosorbent assay
FiO_2_: inspiratory fraction of oxygen
FRC: functional residual capacity
HV_T_: high tidal volume
I:E ratio: inspiratory-to-expiratory ratio
IL-1β: interleukin-1 beta
IL-6: interleukin-6
KC: keratinocyte-derived chemokine
LV_T_: low tidal volume
MCP-1: monocyte chemoattractant protein-1
MV: mechanical ventilation
OI: oxygenation index
p_a_O_2_: partial pressure of oxygen
PBS: phosphate-buffered saline
PEEP: positive end-expiratory pressure
qRT-PCR: quantitative reverse transcription polymerase chain reaction
t_i_: inspiratory time
VILI: ventilator-induced lung injury
V_T_: tidal volume
