# Supplementary material for: Increasing the inspiratory time and I:E ratio during mechanical ventilation aggravates ventilator-induced lung injury in mice
Source: Crit Care. 2015 Jan 28;19(1):23. doi: 10.1186/s13054-015-0759-2 (PMC4336519; doi:10.1186/s13054-015-0759-2)
Supplement: Additional file 1: Table S1. — Providing primer and probe sequences used for qRT-PCR. [file 13054_2015_759_MOESM1_ESM.pdf]

Additional Table 1

Primers used for qRT-PCR

gene	sequence	product length	accession number
MCP-1	forward GGCTCAGCCAGATGCAGTTAA	76 bp	NM_011333
	reverse CCTACTCATTGGGATCATCTTGCT		
	probe FAM-CCCCACTCACCTGCTGCTACTCATTCA-TAMRA		
IL-6	forward CCACGGCCTTCCCTACTTC	147 bp	NM_031168
	reverse TGCACAACTCTTTTCTCATTTCCA		
	probe FAM-TCACAGAGGATACCACTCCCAACAGACCTG-TAMRA		
KC	forward CCGAAGTCATAGCCACACTCAA	74 bp	J04596
	reverse ATTTTCTGAACCAAGGGAGCTT		
	probe FAM-TCGCGAGGCTTGCCTTGACCC-TAMRA		
GAPDH	forward TGTGTCCGTCGTGGATCTGA	76 bp	NM_008084
	reverse CCTGCTTCACCACCTTCTTGA		
	probe FAM-CCGCCTGGAGAAACCTGCCAAGTATG-TAMRA		
IL-1ß	purchased from Applied Biosystems		NP_032387
	Assay no.: Mm00434228		
