# Supplementary material for: Increasing the inspiratory time and I:E ratio during mechanical ventilation aggravates ventilator-induced lung injury in mice
Source: Crit Care. 2015 Jan 28;19(1):23. doi: 10.1186/s13054-015-0759-2 (PMC4336519; doi:10.1186/s13054-015-0759-2)
Supplement: Additional file 2: Figure S1. — Giving mean airway pressure and dynamic compliance measurements under alternating I:E ratios (1:2 and 1:1) during HVT ventilation. [file 13054_2015_759_MOESM2_ESM.pdf]

Additional Fig. 1

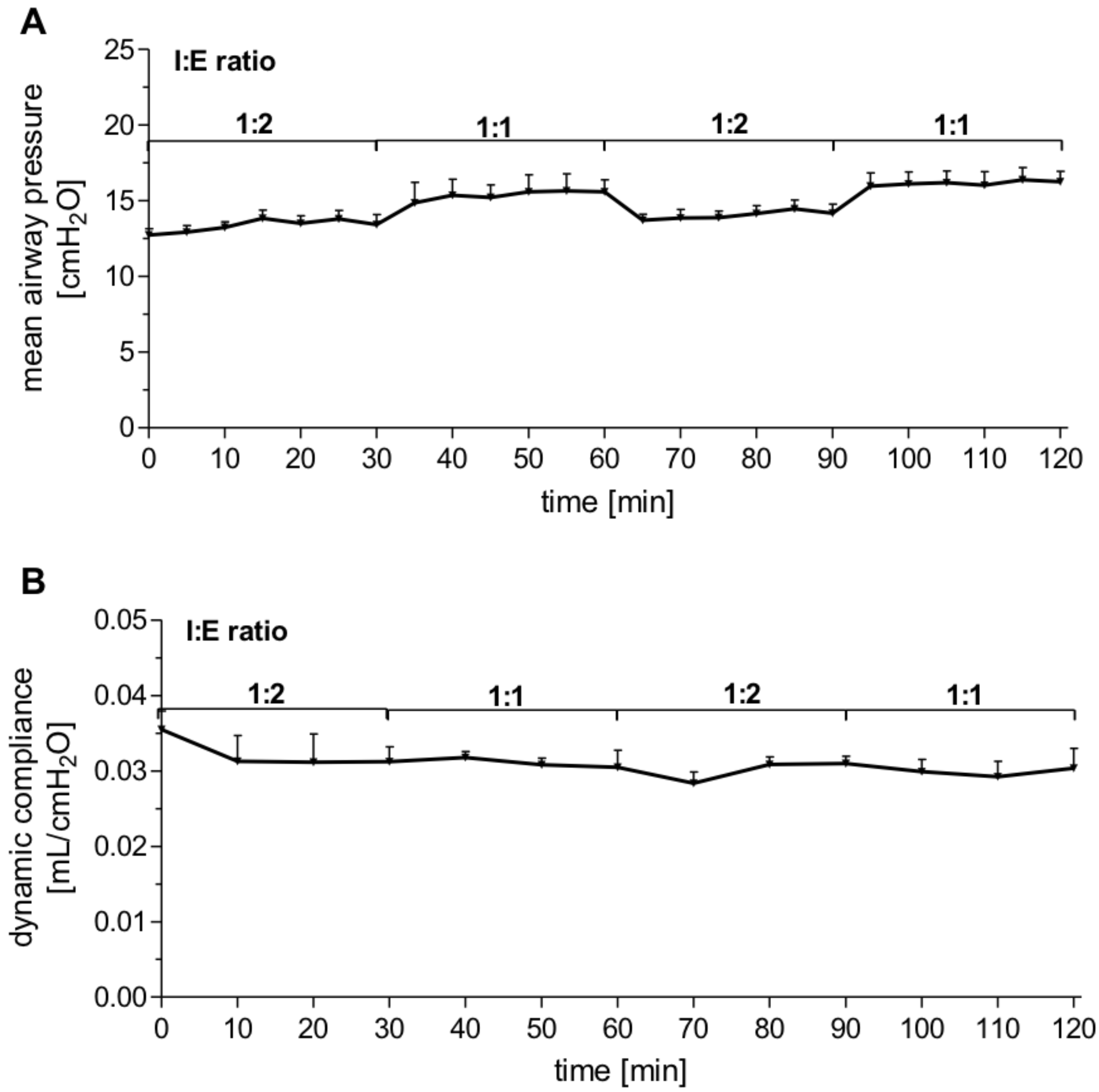

Additional Fig. 1 Increasing inspiratory time and I:E ratio did not result in dynamic hyperinflation

Mice were mechanically ventilated for 2h with high tidal volume (HV_T 34 ml/kg) and an alternating inspiratory : expiratory ratio of 1:2 or 1:1 changed every 30 minutes. Mean airway pressure (A) and dynamic compliance (B) were measured every 10 minutes.
