# Supplementary material for: Increasing the inspiratory time and I:E ratio during mechanical ventilation aggravates ventilator-induced lung injury in mice
Source: Crit Care. 2015 Jan 28;19(1):23. doi: 10.1186/s13054-015-0759-2 (PMC4336519; doi:10.1186/s13054-015-0759-2)
Supplement: Additional file 3: Figure S2. — Providing HE images of all experimental groups. [file 13054_2015_759_MOESM3_ESM.pdf]

Additional Figure 2

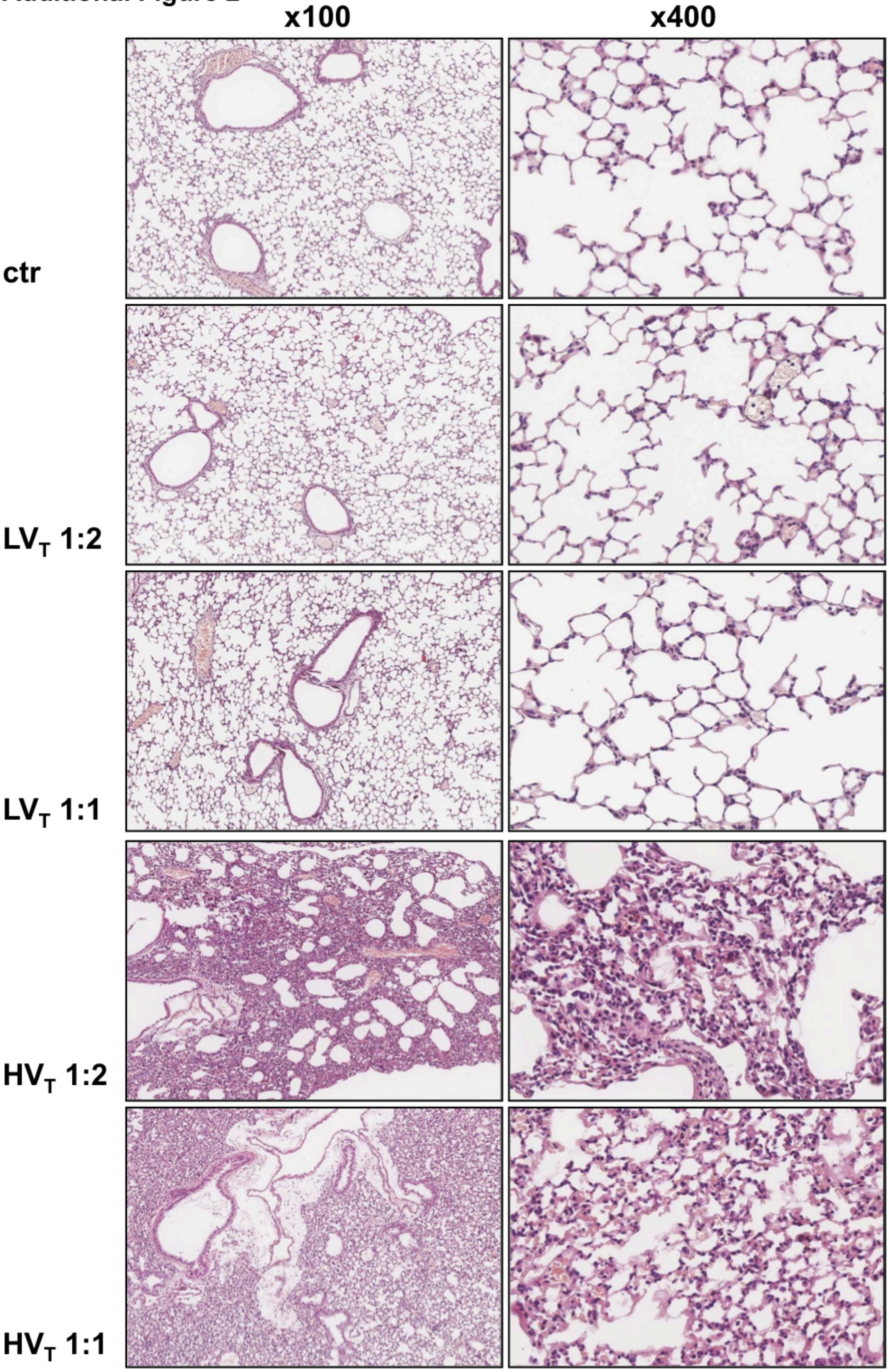

Additional Fig. 2 Histopathologic analysis – HE staining

Mice were mechanically ventilated for 4h with either low tidal volume (LV_T 9 ml/kg) or high tidal volume (HV_T 34 ml/kg) and an inspiratory : expiratory ratio of 1:2 or 1:1, respectively. An alternative endpoint was defined as dropping of mean arterial blood pressure below 40 mmHg, which predicts death with certainty in this model. Controls (ctr) were subjected to LV_T 1:2 ventilation only during operation and were sacrificed before the 4h ventilation protocol started. Paraffin-embedded lung sections were stained with hematotoxilin and eosin. While ctr. and LV_T groups exhibited no signs of lung injury, HV_T 1:2 and 1:1 led to severe leukocyte infiltration of the alveolar wall and tissue collapse, indicative of marked injury to the alveolar walls. Representative images from each group (n=4 each) are shown.
