# Supplementary material for: Increasing the inspiratory time and I:E ratio during mechanical ventilation aggravates ventilator-induced lung injury in mice
Source: Crit Care. 2015 Jan 28;19(1):23. doi: 10.1186/s13054-015-0759-2 (PMC4336519; doi:10.1186/s13054-015-0759-2)
Supplement: Additional file 4: Figure S3. — Providing hemodynamic data. [file 13054_2015_759_MOESM4_ESM.pdf]

Additional Fig. 3

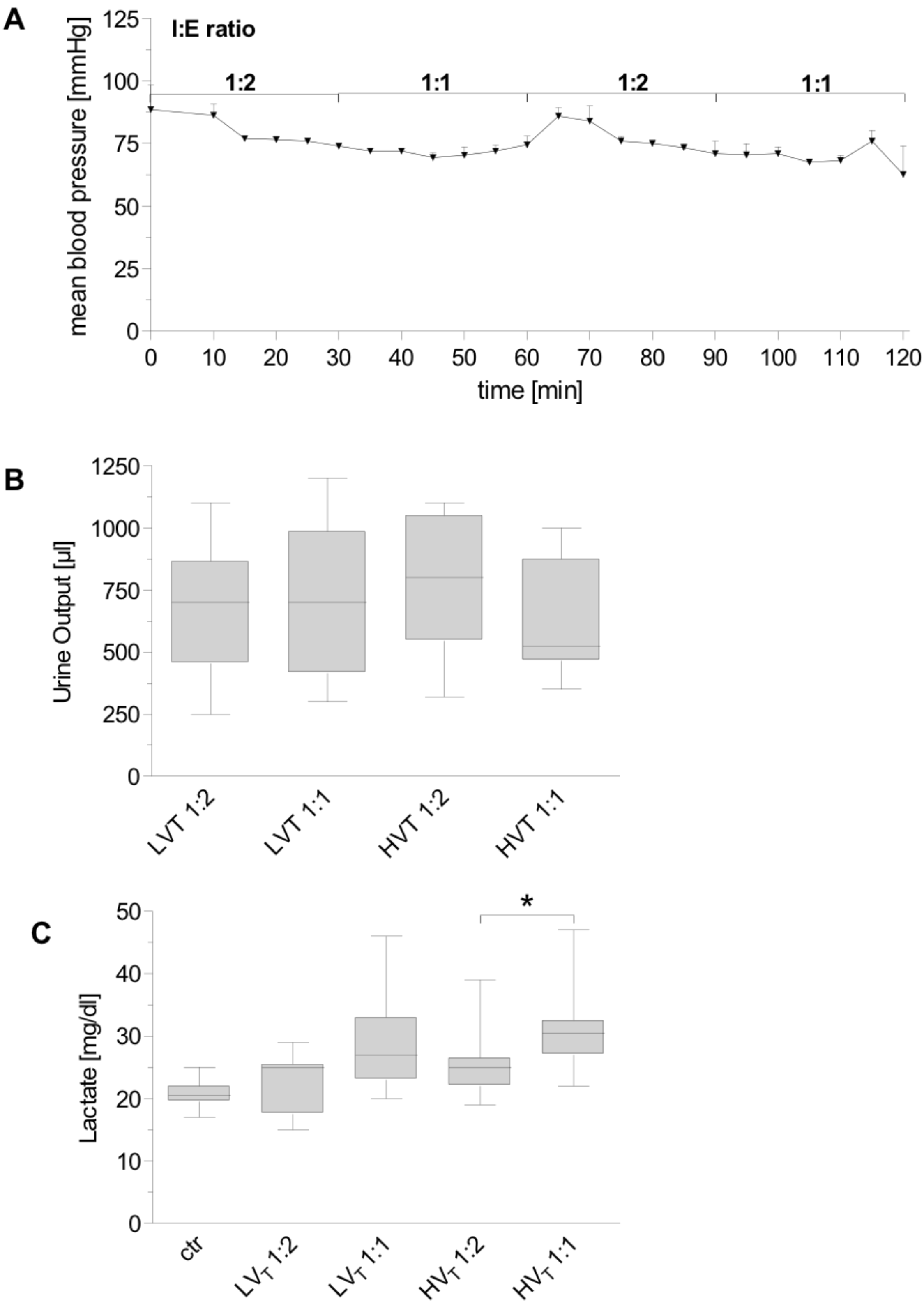

Increasing the inspiratory time and I:E ratio – impact on hemodynamics and markers of tissue perfusion

A Mice were mechanically ventilated for 2h with high tidal volume (HV_T 34 ml/kg) and an alternating inspiratory : expiratory ratio of 1:2 or 1:1 changed every 30 minutes. Mean arterial blood pressure was measured.

B/C Mice were mechanically ventilated for 4h with either low tidal volume (LV_T 9 ml/kg) or high tidal volume (HV_T 34 ml/kg) and an inspiratory : expiratory ratio of 1:2 or 1:1, respectively. An alternative endpoint was defined as dropping of mean arterial blood pressure below 40 mmHg, which predicts death with certainty in this model. Controls (ctr) were subjected to LV_T 1:2 ventilation only during operation and were sacrificed before the 4h ventilation protocol started. Urin output and blood lactate were measured at the end of the experiment.
